# Supplementary material for: Loss of the Novel Mitochondrial Membrane Protein FAM210B Is Associated with Hepatocellular Carcinoma
Source: Biomedicines. 2023 Apr 21;11(4):1232. doi: 10.3390/biomedicines11041232 (PMC10135939; doi:10.3390/biomedicines11041232)
Supplement: Supplementary file 1 [file biomedicines-11-01232-s001.zip › biomedicines-2315429-supplementary.pdf]

Supplementary Material

Supplementary Figure S1

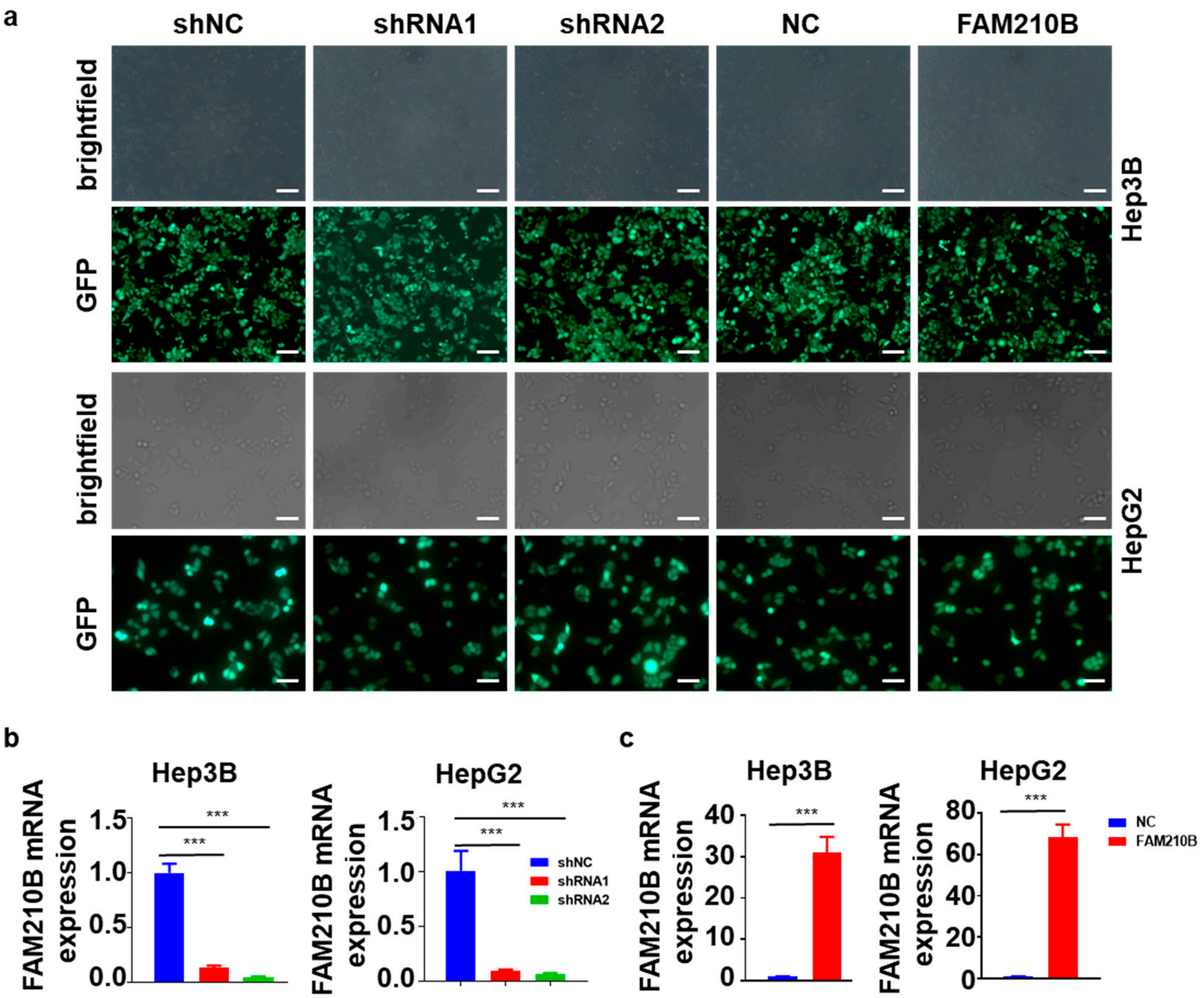

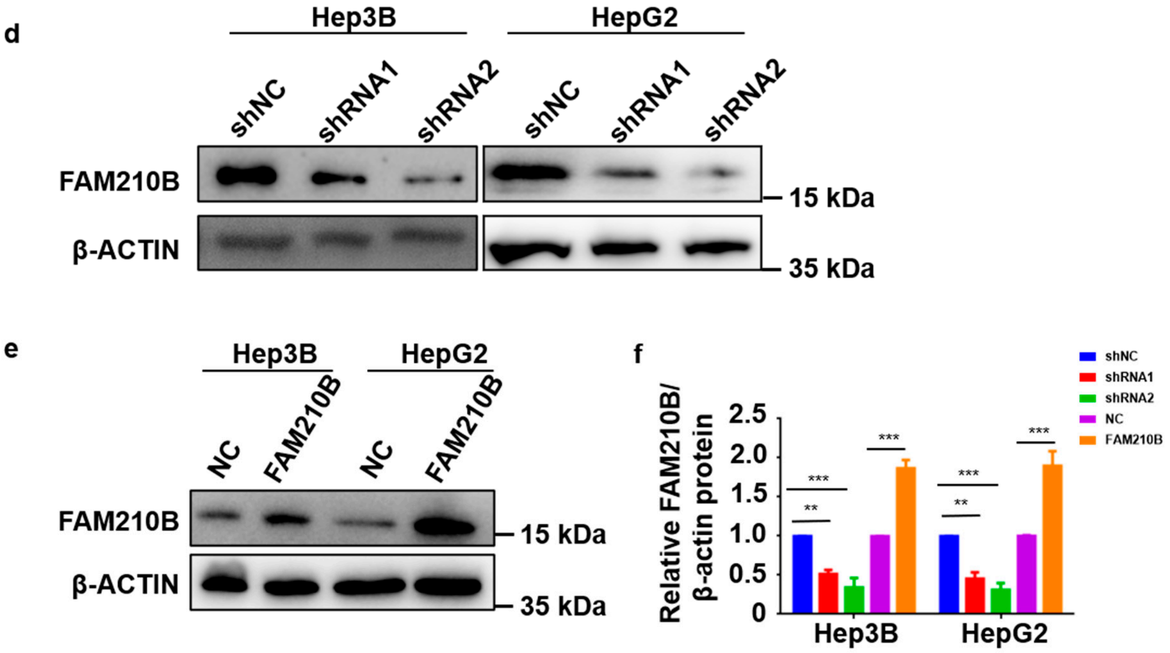

Supplementary Figure S1. Transfection efficiency was measured. (a) The fluorescence microscopy images of cells transfected with lentivirus. (Scale bar=200um). **(b, c)** The FAM210B mRNA levels were determined in Hep3B and HepG2 cells after infection with FAM210B shRNA lentivirus or FAM210B overexpression lentivirus. **(d, e)** The FAM210B protein levels were measured in Hep3B and HepG2 cells following infection with FAM210B shRNA lentivirus or FAM210B overexpression lentivirus. (**f**) Quantitative analysis of FAM210B in Western blot. ** $p < 0.01$; ***$p < 0.001$.

Supplementary Table S1

Characteristics of the cell lines that were used

Cell line	Organism	Tissue and Cell Type	characteristic	Morphology
HL02	human	normal liver	-	Epithelium
Hep3B	human	hepatocellular carcinoma	HBV positive	Epithelium
HepG2	human	Hepatocellular carcinoma	Secrete plasma protein	Epithelium
LM3	human	Hepatocellular carcinoma	High metastasis	Epithelium
Huh7	human	Hepatocellular carcinoma	Secrete alpha fetoprotein	Epithelium
PLC	human	Hepatocellular carcinoma	Secrete HBsAg	Epithelium
